# Harnessing gut microbiota for longevity: Insights into mechanisms and genetic manipulation

**DOI:** 10.1002/imo2.36

**Published:** 2024-10-02

**Authors:** Peng Shi, Simin Xu, Ziling Yang, Liying Wang, Yalun Wu, Ying Li, Zuobin Zhu

**Affiliations:** ^1^ Jiangsu Engineering Research Center for Precision Diagnosis and Treatment of Polygenic Critical Diseases, Key Laboratory of Genetic Foundation and Clinical Application, Department of Genetics Xuzhou Medical University Xuzhou China; ^2^ Department of Biological Sciences, Faculty of Science National University of Singapore Singapore Singapore; ^3^ Xuzhou Key Laboratory of Laboratory Diagnostics, Medical Technology College Xuzhou Medical University Xuzhou China

**Keywords:** aging pathways, gut microbiota, longevity, metagenomic engineering, microbial metabolites

## Abstract

The gut microbiota is pivotal in maintaining health, with most microorganisms being beneficial, except for a few pathogens. Emerging evidence suggests a link between the gut microbiome and aging, hinting at its potential role in longevity. However, understanding the relationship is challenging due to the microbiota's complexity. This perspective summarizes the mechanisms by which gut microbes regulate host lifespan and explores genetic manipulation strategies to promote healthy aging in the elderly.

## Highlights

- This perspective explores the intricate relationship between gut microbiota and host longevity, emphasizing the regulatory mechanisms through which microbial metabolites influence aging pathways.
- It discusses substrates‐based interventions, focusing on microbial‐derived compounds and engineered probiotics to combat chronic inflammation, neurodegenerative disorders, and other age‐related conditions
- It highlights the potential of precise genetic manipulation of gut microbiota through metagenomic engineering as a promising strategy to enhance healthy aging and treat age‐related diseases.

## Graphical abstract

Overview of Gut Microbiota's Role in Aging and Potential for Genetic Manipulation. The relationship between gut microbiota and aging is complex, with significant implications for both health and disease. Advances in genetic manipulation of gut microbiota offer potential interventions to correct dysbiosis, allowing for a microbiome that supports longevity and reduces the risks associated with aging. By targeting specific microbial populations or metabolites, these strategies aim to restore a healthier microbiome, thus promoting healthy aging and mitigating the effects of age‐related diseases.

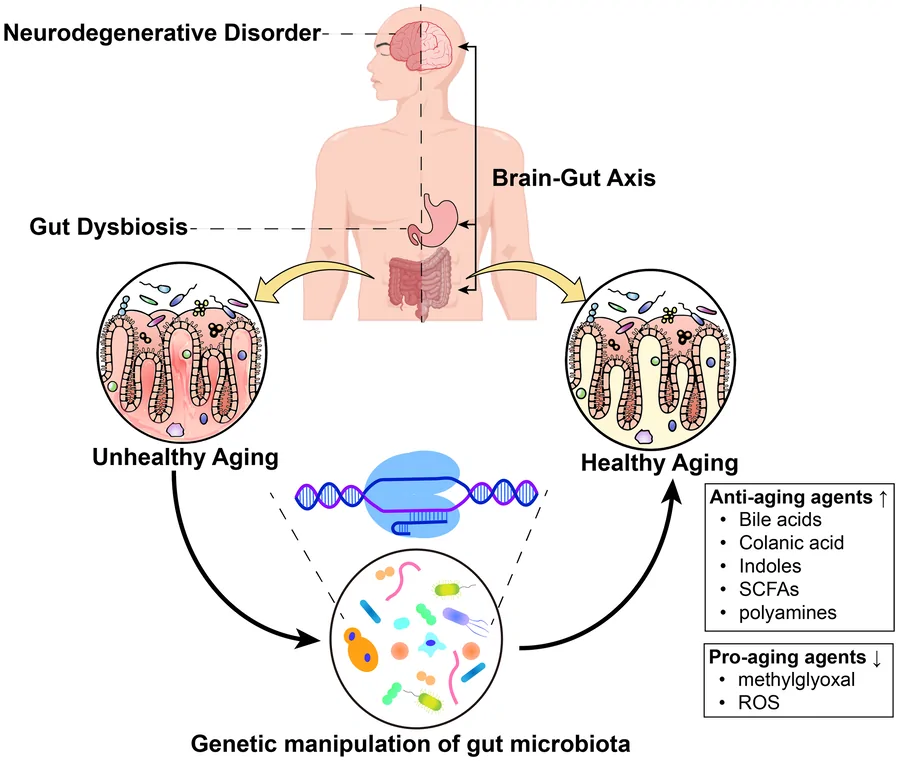

## 1 INTRODUCTION

The intricate relationship between microorganisms and their host is fundamental to many physiological processes in multicellular organisms. A macroscopic host is composed of a vast array of symbiotic microorganisms. Remarkably, the ratio of bacteria to human cells in the adult body is approximately 1:1, and when considering only nucleated human cells, this ratio increases to about 10:1, excluding nonnucleated red blood cells [1]. Advances in technology have significantly enhanced our understanding of host‐microbiome interactions, particularly those involving the gut microbiota, which accounts for 95% of symbiotic microorganisms. The gut microbiota is established at birth and continuously evolves, influenced by various internal and external factors, such as nutrition, lifestyle, sex, and physiology, leading to individual heterogeneity and distinct functions in host.

Recent studies have demonstrated that the gut microbiota exerts its influence on host longevity through a diverse array of regulatory mechanisms [2, 3, 4, 5, 6, 7, 8, 9, 10, 11, 12, 13, 14] (Table 1). Specific patterns of gut microbiota have been associated with extended lifespan, with certain commonalities observed in the gut microbiota of centenarians [15, 16]. For instance, while the gut microbiome composition varies significantly among individuals, a high *Bacteroides* dominance in individuals over 85 years old has been linked to a lower survival rate in the 4‐year follow up [17]. Additionally, gut microbiota is strongly associated with various age‐related diseases, many of which share remarkable similarities with conditions linked to gut dysbiosis‐an imbalance in gut microbiota characterized by changes of their density and composition [18, 19]. Despite these associations, the precise mechanisms by which gut microbiota regulates host longevity remain unclear. This perspective focuses on elucidating the regulatory pathways of longevity associated with gut microbiota, highlighting the critical role of microbe‐derived substances in the complex networks of host signaling pathways.

**Table 1:** Studies on the host‐gut bacteria interaction that regulate the host lifespan

Gut microbiota	Mechanism	Lifespan effect	Microbial metabolites
*L. salivarius* strain FDB89 [2]	Diet restriction dependent	Pro‐longevity (11.9%↑); reduced reproductive capacity, pharyngeal pumping rate and growth	Unknown
*W. koreensis* [3]	Diet restriction dependent, JNK/AMPK/DAF‐16	Pro‐longevity (11%↑); decreased accumulation of lipofuscin; increased locomotor activity	Unknown
*B. subtilis* [4]	Insulin/IGF‐1‐like signaling pathway dependent	Pro‐longevity (14.74%↑); enhanced resistance to environment stress	NO, CSF
*L. rhamnosus* [5]	Insulin/IGF‐1‐like signaling pathway dependent	Pro‐longevity (20%↑); enhanced resistance to oxidative stress	Microbial ligands
*E. coil* K12 mutants [6]	TORC2/SGK‐1/DAF‐16	Pro‐longevity (more than 20%↑)	Methylglyoxal
*E. coil* K12 mutants [7]	Mitochondrial pathways UPR^mt^	Pro‐longevity (40%↑); ameliorate age‐related pathologies of tumor progression and amyloid‐b accumulation	CA
*E. coil* K12 mutants [8]	AHR pathway	Shifted the Kaplan–Meier survival curve to the left; extends Reproductive Span	Indoles
*L. gasseri* SBT2055 [9]	Stress response pathways	Pro‐longevity (37%↑); inhibitation of age‐induced decrease of physiological function including pharyngeal pumping and body bending rates	Immune‐stimulating molecules
*L. fermentum* U‐21 [10]	Keap1‐Nrf2 signaling pathway	Increased the mean lifespan in paraquat treated *C. elegans* and mice; improved resistance of paraquat and preserved dopaminergic neurons	Cell wall peptidases
*L. plantarum* GKM3 [11]	Involved in antioxidative stress	Increased longevity in SAMP8 mice, especially female species, alleviated age‐related cognitive impairment, and reduced oxidative stress	Unknown
*B. animalis subsp. lactis* LKM512 [12]	Inhibited chronic inflammation	Survival rate significantly higher than control after 20 weeks; inhibited constipation, improved maintenance of the intestinal barrier function and suppressed age‐induced inflammatory and oxidative stress responses	PAs
*B. longum* strain BB68 [13]	TIR‐1/JNK‐1/DAF‐16	Pro‐longevity (28%↑)	Cell wall components
*B. infantis* [14]	TIR‐1/PMK‐1/SKN‐1	The extension of lifespan increases as the proportion of BI increases; higher locomotion and decreased accumulation of lipofuscin	Cell wall components

Given the close relationship between gut microbiota and longevity, manipulating gut microbiota emerges as a promising strategy to enhance healthy aging and extend lifespan. Numerous microbiome‐based interventions, including prebiotic and synbiotic supplements, fecal microbiota transplantation (FMT), and health‐linked dietary regimens, have been developed to promote health. However, while these interventions have demonstrated some health benefits, their effectiveness in altering the microbiome to promote health and producing personalized beneficial responses is often limited, depending on the individual's baseline microbiome composition [19]. Hence, instead of focusing solely on traditional microbiome‐based interventions, this perspective explores more precise and efficient strategies toalleviate age‐related diseases and prolong lifespan. These strategies are based on bacteria‐derived substrates and metagenomic engineering of gut microbiota.

## 2 MECHANISMS OF LONGEVITY REGULATION BY GUT MICROBES

The aging process is regulated by an intricate network of signaling pathways, influenced by various genetic and environmental factors. Since the discovery of the first longevity‐regulatory gene, many additional genes have been identified, each contributing to the complex process of aging. A comprehensive perspective has proposed 12 hallmarks of aging, including genomic instability, deregulated nutrient‐sensing, mitochondrial dysfunction, chronic inflammation, dysbiosis and and so on [20]. In this perspective, we categorize the mechanisms by which gut microbes regulate host longevity, focusing on key signaling pathways that link gut microbiota with lifespan extension.

### 2.1 Insulin/insulin‐like growth factor (IGF) signaling (IIS) pathway

The IIS pathway is a well‐known regulator of lifespan, modulating the organism's response to stress, environmental cues, and nutrient availability [21]. This pathway was first discovered in *Caenorhabditis elegans (C. elegans)* over three decades ago, where the knockout of insulin/IGF‐1 transmembrane receptor (IGFR) ortholog DAF‐2 was found to double the lifespan [22]. The conservation of the IIS pathway across species, including *Drosophila melanogaster (D. melanogaster)* and mice, underscores its fundamental role in aging [23]. Moreover, increased intestinal expression of DAF‐16, the *C. elegans* homolog of the human Forkhead box O (FOXO) protein, extends lifespan by promoting DAF‐16 expression in other tissues and inhibiting the IIS pathway [24, 25].

**Figure 1:**
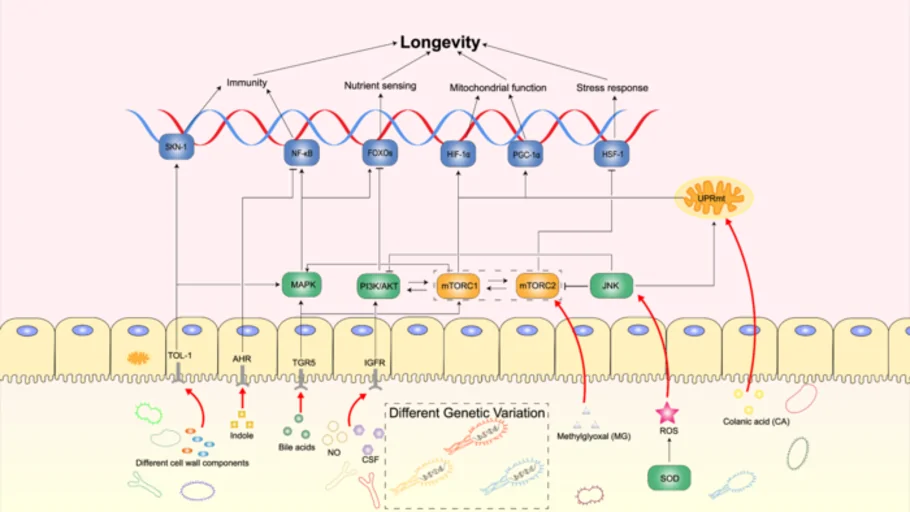
Mechanisms of longevity regulation by gut microbes. Gut microbiota influences host lifespan through a complex network of signaling. Key pathways include the Insulin/IGF Signaling Pathway (IIS), where gut‐derived metabolites like nitric oxide (NO) and quorum‐sensing pentapeptide (CSF) interact with the IGF receptor (IGFR) ortholog DAF‐2, modulating the PI3K/AKT/mTOR pathway to affect nutrient sensing and stress response. The TOR signaling pathway is influenced by the bacterial metabolite methylglyoxal (MG), which inhibits mTORC2, activating longevity‐promoting factors such as DAF‐16, the *C. elegans* homolog of the human FOXO protein. Mitochondrial function and biogenesis are regulated by gut microbiota through metabolites like colanic acid (CA), which induces mitochondrial fission and activates the mitochondrial unfolded protein response (UPR^mt^), enhancing mitochondrial health and longevity. Stress response pathways are modulated by gut‐derived small molecules such as indole, which activate receptors like the aryl hydrocarbon receptor (AHR) to maintain ROS homeostasis and extend lifespan. Additionally, immune pathways, including MAPK and NF‐κB, are regulated by microbial metabolites such as bile acids and cell wall components, controlling chronic inflammation and promoting longevity.

The gut microbiota can influence the IIS pathway through the production of specific metabolites. For instance, *C. elegans* lacks nitric oxide synthases (NOS) to produce nitric oxide (NO), yet NO produced by gut bacteria can extend the worm's lifespan in a DAF‐16‐ and HSF‐1‐dependent manner [26]. Further study demonstrated that colonization by *Bacillus subtilis* NCIB3610, which produces the quorum‐sensing pentapeptide Competence Sporulation stimulating Factor (CSF) and NO, extends *C. elegans* lifespan through the DAF‐16‐dependent IIS pathway [4]. However, due to genetic variation, not all strains of *Bacillus subtilis* can produce CSF. Similarly, the *Lactobacillus rhamnosus* CNCM I‐3690 strain increases the lifespan of *C. elegans* via the DAF‐2/DAF‐16‐dependent IIS pathway by promoting DAF‐16 transcription and increasing oxidative resistance, although the exact mechanisms remain unclear [5] (Figure 1).

### 2.2 TOR signaling pathway

The target of rapamycin (TOR) is a serine/threonine protein kinase that is crucial in regulating various metabolic processes, including protein synthesis, glucose metabolism, and mitochondrial function. TOR exists in two complexes, TORC1 and TORC2, and responds to both environmental and hormonal signals, especially nutritional cues [27]. The TOR pathway is recognized as a key molecular mechanism downstream of IIS pathway, particularly in the context of diet restriction, which has been associated with lifespan extension [28]. Inhibition of TOR has been shown to enhance longevity across different species by improving stress resistance, promoting autophagy, enhancing mitochondrial function, supressing inflammation, and preserving stem cell population [27, 29, 30, 31, 32]. A recent genome‐wide screening of the *Escherichia coli K‐12* showed that the reduction of the bacteria‐derived metabolite methylglyoxal (MG) extends host longevity by inhibiting TORC2 and subsequently activating DAF‐16 [6] (Figure 1). This highlights the role of gut microbes in modulating TOR signaling, which influences host lifespan through the production of specific metabolites.

### 2.3 Mitochondrial pathways

Mitochondria are integral to the aging process, with mitochondrial dysfunction recognized as one of the hallmarks of aging [33, 34]. Research has shown a complex interplay between mitochondria and the nucleus in controlling aging [35, 36]. During aging, increased acetylation levels can inactivate key genes such as PGC‐1α and HIF‐1α, which are crucial for mitochondrial function and biosynthesis, leading to mitochondrial damage [37]. Maintaining a balance between mitochondrial synthesis and mitophagy is essential for preserving cellular homeostasis and promoting longevity [38]. Thus, dynamic modulation of mitochondrial function offers a promising approach to extend lifespan.

Recent studies have underscored the role of mitochondrial stress response, particularly the mitochondrial unfolded protein response (UPR^mt^), in regulating longevity by promoting mitochondrial biogenesis, function and dynamics [35, 39]. Additionally, there is a growing body of evidence supporting a complex communication network between gut microbiota and host mitochondria [40]. For instance, enterobactin, an iron carrier produced mainly by enterobacteria, is absorbed and transported into the host mitochondria, where it interacts with ATP synthases to regulate host development [41, 42].

In another study, a novel longevity‐promoting mechanism associated with UPR^mt^ was discovered through a genome‐wide screen of *E. coli* K12 [7]. the overproduction of colanic acid (CA), a polysaccharide secreted by many enterobacterial species, was found to induce mitochondrial fission and enhance UPR^mt^ under stress conditions, thereby extending the lifespan of the host [7] (Figure 1). These findings suggest that gut microbes can influence host longevity through modulating specific signaling pathways associated with mitochondria and their metabolites.

### 2.4 Stress response pathways

Stress resistance is a crucial factor in regulating the lifespan of an organism, enabling it to cope with various environmental and physiological stressors, such as high temperature, UV irradiation, pathogens, and reactive oxygen species (ROS). Sublethal exposure to these stressors is believed to activate protective responses that can enhance lifespan [43]. For example, *C. elegans* exposed to oxidative stress at an early stage exhibits a better resistance to heat shock and a longer lifespan compared to unstressed worms [44]. Moreover, key longevity‐related pathways, such as IIS and mammalian TOR (mTOR) pathway, regulate the expression of stress resistance genes such as *hsf‐1*, *jnk‐1*, and *skn‐1* [24].

ROS are produced not only by the mitochondrial electron transport chain but also by external factors through NADPH oxidases (NOXs) and signaling pathways such as the IIS pathway [45]. While high levels of ROS can damage proteins, DNA, and cells, maintaining ROS homeostasis is essential for organism growth and aging [45, 46]. The gut microbiota can significantly influence ROS homeostasis and stress pathways. For instance, both live and dead forms of the *Lactobacillus gasseri* strain SBT2055 (LG2055) have been shown to extend the lifespan of *C. elegans* by maintaining low ROS levels through increased activity of superoxide dismutase (SOD), a major component of the antioxidant defense system. This strain also upregulates the expression of stress response genes such as *skn‐1*, *trx‐1*, *clk‐1* and *hsp‐70* [9, 46]. Similarly, supplementation with the *L. fermentum* strain U‐21 promotes longevity in both *C. elegans* and a mouse model of Parkinson's disease by enhancing resistance to oxidative stress and preserving the dopaminergic neurons respectively [10]. Furthermore, indole, a small molecule produced by *E. coli* K‐12 variant, has been was found to enhance stress resistance, lifespan, and the reproductive span via the aryl hydrocarbon receptor (AHR), a conserved detector of xenobiotic small molecules, in *C. elegans*, *D. melanogaster* drosophila, and mice [8] (Figure 1). These findings suggest that probiotic supplementation and other microbial interventions may be promising strategies for maintaining ROS homeostasis and promoting healthy aging.

### 2.5 Immunity pathways

The immune response is a sophisticated mechanism that protects the host from a variety of pathogens while also playing a crucial role in inflammation and tissue repair. However, despite its benefits in pathogen clearance, chronic immune activation and inflammation‐often referred as “inflammaging”‐can contribute to the aging process and age‐related diseases. Immunity is thus a vital factor in modulating longevity, and weaker immune defenses have been identified as a hallmark of aging [33, 47]. The mitogen‐activated protein kinase (MAPK) signaling pathway, which is conserved across eukaryotes, lies at the core of immune responses and inflammation [48, 49, 50]. Chronic inflammation is involved in aging and age‐related diseases and may persist throughout the lifespan of an organism, affecting healthy living [51].

Intestinal symbiotic microbiota can regulate host lifespan through their effects on immune responses. A direct example of this is the oral administration of recombinant lactic acid bacteria (LAB) *Escherichia coli* Nissle 1917 (ECN) that produce SOD and catalase (CAT). This intervention has been shown to inhibit inflammation in the gastrointestinal tract, induced by agents like colonic dextransodium sulfate (DSS), 2,4,6‐trinitrobenzene sulfonic acid (TNBS), and oxazolone‐induced inflammation in gastrointestinal tract [52], thereby potentially extending lifespan. In addition, the *Bifidobaceterium lactis* strain LKM512, a known probiotic, has been proven to suppress chronic inflammation, maintain intestinal barrier integrity, and extend the lifespan of mammals by increasing the concentrations of intestinal polyamines (PAs) [12]. Polyamines are known to play a significant role in cellular growth and function, which is critical for maintaining gut health and longevity [53].

Another vital microbial metabolite in the intestinal is bile acids (BAs), which have been reported to play a crucial role in the aging process. A study involving 160 centenarians found that these individuals have a distinct gut microbiome enriched with microbes capable of generating unique secondary BAs [54]. BAs exert antiaging effect through two primary mechanisms: directly combating pathogens and modulating immune responses [54, 55]. For one thing, BAs have potent antibacterial effects against gram‐positive pathogens, including multidrug‐resistant strains, and play a significant role in maintaining intestinal barrier integrity and reducing inflammation [54]. For another, BAs act as signal molecules, activating immune pathways through nuclear and membrane receptors such as Takeda G‐protein receptor 5 (TGR5) and pregnane X receptor (PXR), also known as the steroid and xenobiotic‐sensing receptor [55]. Activation of TGR5 by BAs can trigger MAPK signaling via ERK1/2 and mTOR, leading to reduced cytokine transcription and macrophage migration [56]. On the other hand, PXR activation controls the feedback regulatory pathway of the BA pool size and composition, which is crucial for immune modulation [55]. For example, the deletion of PXR leads to a decrease in the BA pool, especially the cholic acid, which is converted to lithocholic acid and involved in immune responses as previously mentioned [54, 57]. Interestingly, activation of PXR by synthetic ligands has been shown to inhibit the transcription of inflammatory genes such as interleukin‐1 beta (*IL1B)* and nitric oxide synthase 2 *(NOS2)* by binding to the NF‐κB binding sites in the gene promoters [58].

Moreover, bacterial cell wall components can activate immune responses in the host and regulate lifespan. For instance, the cell wall component of the *Bifidobacterium longum* (*B. longum) strain BB68 and Bifidobacterium infantis (B. infantis)* have been reported to extend the host lifespan in *C. elegans* by activating the p38 MAPK pathway through Toll‐like receptor homolog (TOL‐1) [13, 14, 59]. Another notable finding is that a reduced abundance of immune‐stimulating bacteria can protect mice from neurodegenerative disorders induced by C9ORF72 mutations and systemic inflammation, providing further evidence that gut microbes influence inflammation and lifespan [60, 61].

These examples illustrate the profound impact that gut microbiota and their metabolites can have on the immune system and overall longevity, emphasizing the potential of microbiome‐targeted therapies in promoting healthy aging.

## 3 SUBSTRATES‐BASED INTERVENTIONS TO TREAT AGE‐RELATED DISEASES

Aging is characterized by alterations in gene expression, proteostasis, and cellular function, leading to a pathophysiological decline in most organs and an increased susceptibility to various diseases, including chronic inflammation, neurodegenerative disorders, and cancer [33, 62]. Changes in the gut microbiota during aging are closely linked to these age‐related diseases, especially those associated with gut dysbiosis, which can exacerbate chronic inflammation and neurodegenerative conditions [18, 19].

The beneficial components of the gut microbiota, along with their secreted substances, play a crucial role in maintaining the integrity of the intestinal barrier. This barrier is essential for preventing gut dysbiosis‐induced chronic inflammation and reducing the risk of cancer [63]. Several studies have demonstrated that microbe‐derived substances, such as CA, PAs, indole and its derivative indole‐3‐lactic can inhibit chronic inflammation, promote the integrity of the intestinal barrier, and ameliorate the progression of intestinal tumors [7, 12, 64]. Moreover, innovative approaches, such as the development of engineered probiotic, have shown promise in treating inflammatory conditions. For instance, an engineered yeast probiotic combining a human P2Y2 G protein‐coupled receptor (GPCR) and an extracellular ATP (eATP)‐degrading enzyme has been designed to monitor and regulate eATP levels, effectively treating inflammatory bowel disease [65]. These findings highlight the potential of microbe‐derived substances and engineered probiotics in preventing and treating gut dysbiosis related diseases.

Besides chronic inflammation, the relationship between gut microbiota and neurodegenerative diseases has emerged as a particularly area of research. The microbiota‐gut‐brain axis, which encompasses bidirectional communications between gut microbe and the brain, plays a critical role in this context [66, 67]. Germ‐free mice exhibit significant differences in brain development, function, and behavior compared with their colonized counterparts, underscoring the importance of gut microbiota in neurological health [68]. Studies have shown that the probiotic strain *L. plantarum* GKM3 may promote longevity, reduce oxidative stress, and alleviate cognitive impairment associated with aging, further emphasizing the role of gut microbes in age‐related neurological diseases [11].

**Figure 2:**
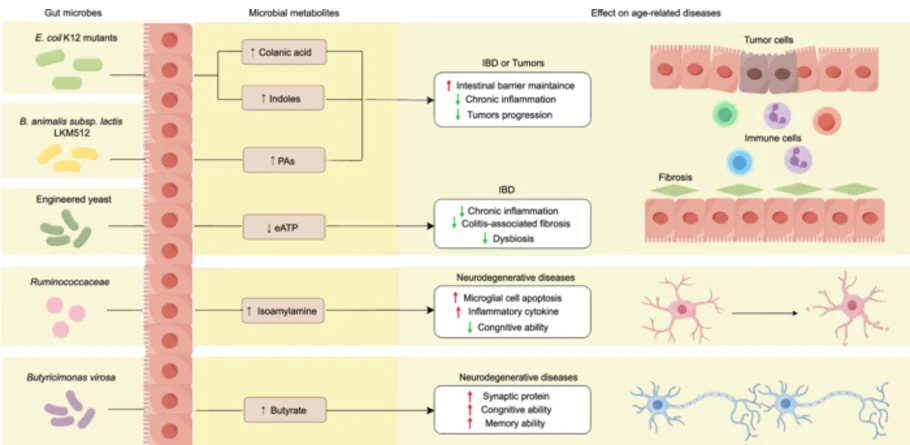
Substrates‐based interventions to treat age‐related diseases. Gut microbiota and its derived substrates play a critical role in influencing age‐related diseases, including chronic inflammation, neurodegenerative disorders, and cancer. This schematic highlights the beneficial effects of microbe‐derived compounds, such colanic acids (CAs), indoles and polyamines (PAs), on the integrity of the intestinal barrier and their ability to reduce chronic inflammation. Engineered probiotics designed to modulate extracellular ATP (eATP) levels are shown to ameliorate inflammatory bowel disease (IBD). In addition, two mechanisms linking gut microbiota to neurodegenerative diseases are depicted: (1) gut microbiota from the *Ruminococcaceae* family produce isoamylamine (IAA), which increases microglia apoptosis, contributing to cognitive decline, and (2) high‐salt diets reduce *Butyricimonas virosa* abundance, impairing synaptic function through the PI3K‐Akt pathway.

Moreover, multiple reviews have summarized the influence of gut microbiota composition and the production of bacterial metabolites, such as short‐chain fatty acids (SCFAs) and other bioactive compounds [66, 69]. While evidence strongly supports therole of gut microbiota in these conditions, the precise mechanisms remain unclear. However, recent studies have shed light on two such mechanism: the production of isoamylamine (IAA) by gut microbiota belonging to the *Ruminococcaceae* family was found to increase microglia apoptosis via modulation of S100A8 expression, contributing to cognitive decline in aged mice. Remarkably, targeting *Ruminococcaceae* with specific phages or using a single strain DNA oligonucleotide (S100p1‐G) reversed cognitive dysfunction in these mice by blocking IAA production [70]. Another mechanism is high‐salt diet results in a decrease in the abundance of *Butyricimonas virosa* which could metabolize fiber into butyrate and influenced synapses and memory through the synaptic protein synapsin I (SYN1) via the PI3K‐AKT pathway [71]. These studies provide a precise understanding of how gut microbiota can influence age‐related brain dysfunction and suggests potential therapeutic strategies for preventing neurodegenerative diseases through microbiota modulation (Figure 2).

Given the critical role of gut microbiota in longevity and the significant clinical potential for treating age‐related diseases, it is imperative to further understand the precise mechanisms by which gut microbes regulate host longevity and health. This understanding will pave the way for more targeted and effective interventions aimed at promoting healthy aging.

## 4 METAGENOMIC ENGINEERING OF GUT MICROBIOTA: MANIPULATING GUT MICROBIAL GENETIC VARIATION TO PROLONG HOST LONGEVITY

Genetic variation within the gut microbiome is a crucial factor influencing host longevity. This variation is fundamental to natural selection and diversity within biological systems [72]. In mammals, the gut microbiome is not only heritable but also highly susceptible to environmental factors such as diet, medication, and lifestyle [73]. These environmental influences can drive alterations in the microbiome, leading to the acquisition of new traits such as antibiotic resistance, virulence, or the ability to produce substances that promote host longevity [74]. The adaptive nature of the gut microbiota implies that genetic variants conferring benefits to the host are more likely to be propagated and preserved.

**Figure 3:**
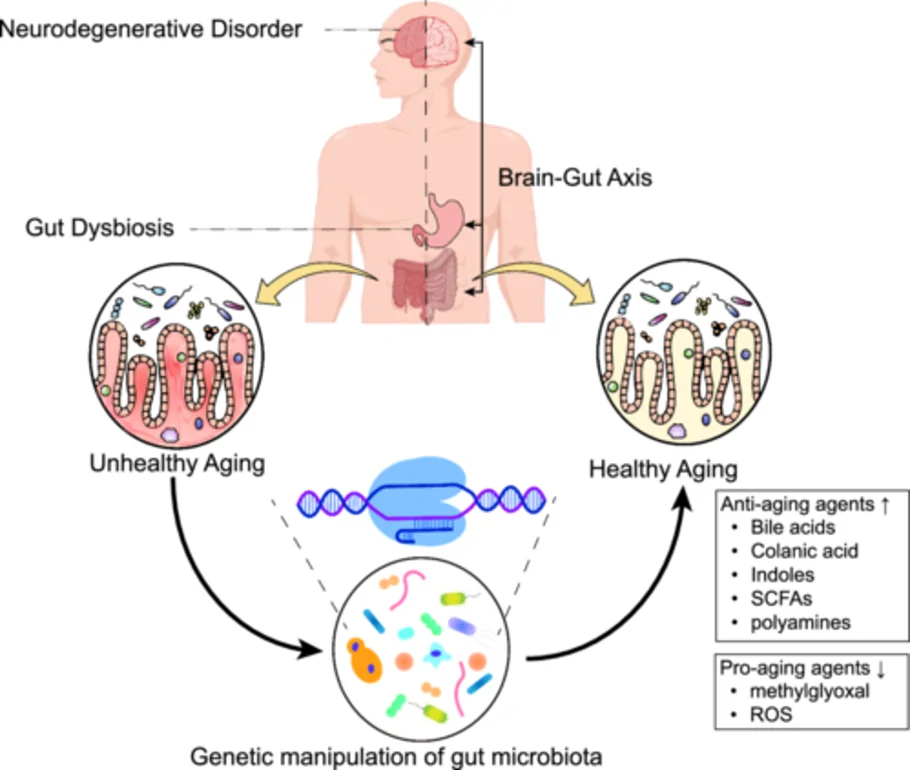
Overview of gut microbiota's role in aging and potential for genetic manipulation. The relationship between gut microbiota and aging is complex, with significant implications for both health and disease. Gut dysbiosis, an imbalance in microbial composition, is linked to unhealthy aging, characterized by chronic inflammation and a heightened risk of neurodegenerative disorders. On the negative side, dysbiosis contributes to age‐related diseases by disrupting normal physiological processes, particularly those related to immune and neurological function. In contrast, a balanced gut microbiota plays a protective role, promoting healthy aging and resilience against age‐related conditions. A key element in this balance is the brain‐gut axis, which facilitates bidirectional communication between the gut and the central nervous system, influencing both cognitive health and overall physiological aging. Advances in genetic manipulation of gut microbiota offer potential interventions to correct dysbiosis, allowing for a microbiome that supports longevity and reduces the risks associated with aging. By targeting specific microbial populations or metabolites, these strategies aim to restore a healthier microbiome, thus promoting healthy aging and mitigating the effects of age‐related diseases.

While numerous studies have explored the relationship between microbiome composition—specifically species and strain diversity—and host longevity, less attention has been given to the impact of genetic variations within these microbes [75, 76]. As more mechanisms by which beneficial gut microbes promote health and extend lifespan are uncovered, specific enzymes and substrates derived from microbiome have been identified as key players in gut microbiota‐host interactions [6, 7, 16, 42]. Compared to broad manipulations of species and strain diversity, targeting genetic variations that influence the production of these beneficial compounds may offer a more efficient and precise approach to promoting longevity (Figure 3).

Metagenomic engineering of the gut microbiota represents a promising strategy for manipulating these genetic variations. This approach could serve as a powerful tool in treating age‐related diseases and extending lifespan by directly influencing the genetic makeup of the gut microbiome. Recent advances in metagenomic engineering techniques, particularly those involving mobiles genetic elements (MGE) and the CRISPR‐Cas system have opened new avenues for this type of intervention.

Prokaryotic MGE includes 4 major classes which are plasmids, prophages, DNA transposons, and intervening sequence elements [77]. Recently, several potent metagenomic engineering platforms have been developed. These include.

### 4.1 MAGIC (Metagenomic Alteration of Gut Microbiome by In Situ Conjugation)

**Figure 4:**
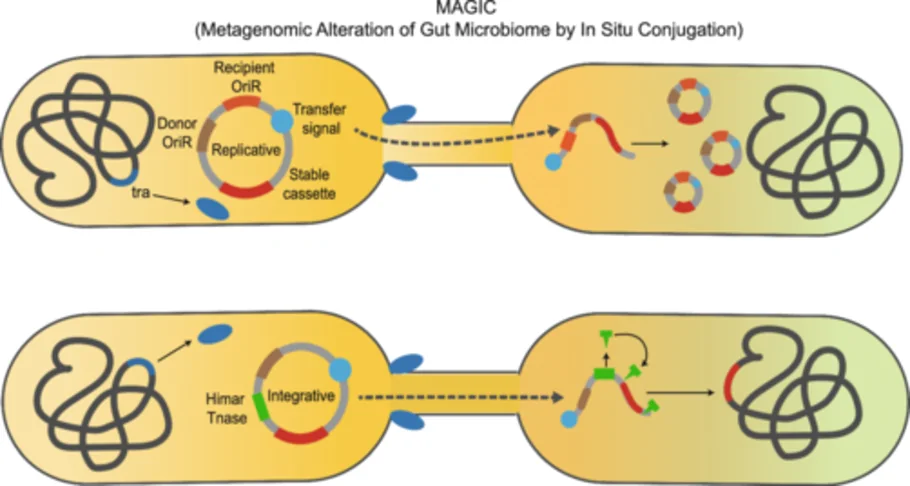
Metagenomic Alteration of Gut Microbiome by In Situ Conjugation (MAGIC). MAGIC is a platform, designed for the genetic modification of gut microbiota directly in their native environment via horizontal gene transfer. It utilizes engineered *Escherichia coli* strains as donor bacteria to transfer replicative or integrative genetic payloads into diverse microbial recipients in the gut microbiome. In the top panel, the donor bacterium contains a replicative vector, marked by the origin of replication (OriR), a stable genetic cassette, and conjugative machinery (tra). The conjugation process enables the transfer of the replicative vector from the donor to the recipient bacterium, where the genetic material replicates independently, thereby allowing stable expression of the introduced gene in the recipient population. In the bottom panel, the donor bacterium carries an integrative genetic vector with a transposase (Himar Tnase), which facilitates the integration of the desired genetic payload into the recipient's chromosome.

MAGIC is a platform designed to genetically modify gut microbiota directly within their native habitat through horizontal gene transfer. This approach enables the transfer of desired genetic payloads into diverse microbial taxa in the gut microbiome without the need to isolate and cultivate the bacteria in a lab setting. MAGIC leverages bacterial conjugation mechanisms, specifically using engineered *Escherichia coli* strains as donors to introduce replicative or integrative genetic vectors into recipient bacteria within a complex microbial community, such as the mammalian gut (Figure 4). This platform's primary advantage lies in its ability to deliver engineered genes to a broad range of bacterial recipients in their native habitat, bypassing the need for isolation and cultivation in laboratory conditions, which is often difficult for many microbial species. This approach allows for the study of microbiota in their natural states, maintaining complex ecological interactions and improving the ecological relevance of the modifications. Furthermore, MAGIC can target a broad range of bacteria by utilizing vectors with broad‐host‐range replication or transposon integration systems, making it a versatile tool for microbiome engineering. Its rapid modification capability (within 6 h) and the persistence of transconjugant donor bacteria for at least 15 days highlight its efficiency for short‐term genetic manipulation. However, maintaining the stability of the introduced genetic payloads in vivo can be difficult, as they may be lost over time due to negative selection or instability within the microbial population. Additionally, its applicability is currently limited to modifying approximately 5% of the gut microbiome, the method may require further optimization for efficient long‐term retention of the genetic modifications and might have limited control over the precise targeting of specific species within a complex community [78].

### 4.2 Phage‐delivered CRISPR‐Cas9

**Figure 5:**
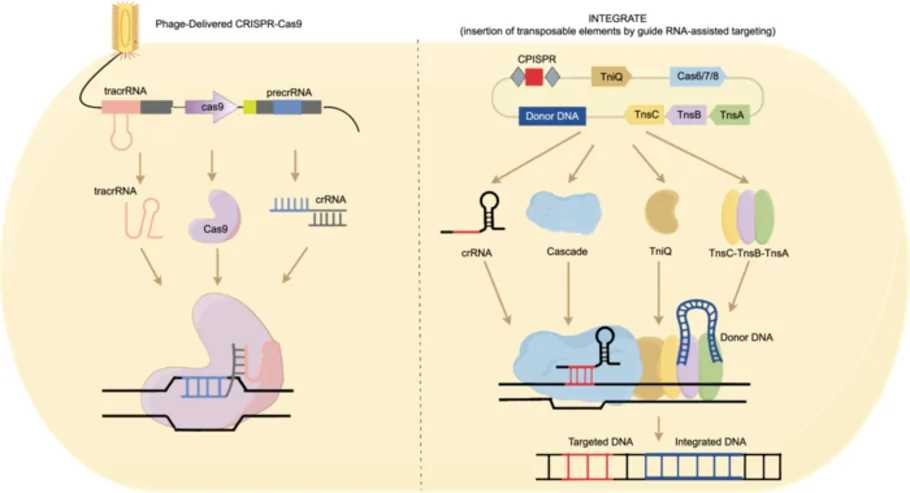
Phage‐delivered CRISPR‐Cas9 and INTEGRATE (insertion of transposable elements by guide RNA‐assisted targeting). This figure illustrates two complementary genome editing technologies: Phage‐delivered CRISPR‐Cas9 and INTEGRATE. Phage‐delivered CRISPR‐Cas9 (left panel) leverages engineered filamentous phages, such as M13, to introduce the CRISPR‐Cas9 system into specific bacterial strains within the gut microbiome, which targets and induces sequence‐specific double‐strand breaks (DSBs) in the bacterial genome, leading to strain‐specific depletion or genomic deletions. INTEGRATE (right panel) is a CRISPR‐based system designed for the precise insertion of kilobase‐sized DNA sequences into bacterial genomes via transposable elements. The system uses the CRISPR Cascade complex to guide TniQ and the transposase complex (TnsA, TnsB, TnsC) to specific genomic sites, allowing the insertion of donor DNA without the need for DSBs or homologous recombination.

Phage‐delivered CRISPR‐Cas9 is a technology that utilizes engineered bacteriophages to deliver CRISPR‐Cas9 systems to specific bacterial strains within the gut microbiome. This method leverages filamentous phages, such as M13, which can transfer genetic material to bacteria without causing cell lysis. By incorporating a CRISPR‐Cas9 system into the phage, researchers can target and induce strain‐specific depletion or genomic deletions in bacterial populations within the gut. The system enables precise gene editing, allowing for the removal or modification of specific bacterial strains or genes (Figure 5). It offers several advantages for microbiome manipulation, particularly in enabling strain‐specific targeting and genetic editing within complex microbial communities, such as the gut. Its precision allows for the depletion or genomic modification of specific bacterial strains without affecting the broader microbiome, making it a powerful tool for studying bacterial functions or developing therapeutic interventions. Additionally, it can be used without introducing exogenous bacteria, which can be challenging in other microbiome editing techniques. However, this method also has some limitations. One major challenge is the potential for bacteria to evade CRISPR‐Cas9 targeting, either by losing the CRISPR array or through mutations at the target site, which can result in incomplete depletion. Furthermore, the efficiency of phage delivery in vivo can be limited due to factors such as phage survival during gastrointestinal transit or the requirement for antibiotic selection to maintain genetic modifications, which can disrupt the microbiome. Overall, while phage‐delivered CRISPR‐Cas9 is a promising technology, further optimization is needed to enhance targeting efficiency and overcome bacterial escape mechanisms [79, 80].

### 4.3 INTEGRATE (Insertion of Transposable Elements by Guide RNA‐Aassisted Targeting)

INTEGRATE is a CRISPR‐based system that enables highly efficient and precise insertion of kilobase‐sized DNA sequences into bacterial genomes. This system utilizes a CRISPR‐Cas mechanism to guide transposons to specific genomic locations, where DNA can be integrated without requiring double‐strand breaks (DSBs) or homologous recombination (HR). INTEGRATE leverages transposition proteins, such as TnsA, TnsB, and TnsC, along with the RNA‐guided DNA targeting complex (TniQ‐Cascade), to achieve programmable and marker‐free genomic integration with ~100% efficiency in bacteria (Figure 5). INTEGRATE offers several advantages in bacterial genome engineering, particularly its ability to achieve highly efficient, precise, and programmable insertion of large DNA sequences without requiring DSBs or HR. This system allows for seamless, marker‐free integration of up to 10 kilobases of DNA at specific genomic sites, offering high fidelity and flexibility in multiplexed gene insertions by using CRISPR arrays with multiple guide RNAs. Another key advantage is its broad host‐range activity, making it useful across diverse bacterial species and within complex microbiomes. Additionally, INTEGRATE avoids the cytotoxic effects associated with DSBs and offers rapid, efficient insertion, even in non‐model organisms. However, INTEGRATE has some limitations. It cannot achieve scarless editing or point mutations, which require other gene‐editing technologies. Furthermore, its reliance on transposon end sequences may introduce sequence constraints, and the system may be less suited for applications where precise control over the insertion of smaller or functional sequences is needed [81].

The optimal utilization of these cutting‐edge microbiome‐based genetic techniques holds significant potential to revolutionize the treatment of age‐related diseases. By leveraging these tools, we can move closer to breakthrough in promoting healthy aging, ultimately improving the quality of life and extending lifespan.

## 5 CONCLUSION

The studies discussed in this perspective highlight a novel approach centered on the metabolites produced by gut microbiota, demonstrating the potential of utilizing these microorganisms to maintain health and extend lifespan. This approach holds significant promise for transforming pharmaceutical and medical science by introducing new concepts for treating age‐related conditions. There is now substantial evidence indicating that the dynamic interaction between the host and its microbiota influences many physiological processes, including those related to aging. However, the specific role of genetic variations within gut microbes in extending lifespan remains largely unexplored.

We propose that microbe‐derived metabolites, produced as a result of specific genetic variations within gut microbes, play a crucial role in modulating the aging process and maintaining health by transmitting longevity signals through complex pathways. Despite the promising potential, understanding the intricate compositions of gut microbiota, its genetic variations, and the diversity of metabolites remains a formidable challenge. Focusing on the identification and functional assessment of specific microbial metabolites through high‐throughput screening could provide a promising pathway for developing effective drugs aimed at enhancing host health and promoting a healthy lifespan.

Moreover, advancements in gut microbial gene editing techniques present an exciting frontier in the development of more precise and feasible therapeutics to combat age‐related diseases and promote longevity. By harnessing these cutting‐edge technologies, we can envision a future where targeted manipulation of the gut microbiome becomes a cornerstone in the pursuit of healthier aging and extended lifespan.

## AUTHOR CONTRIBUTIONS

Zuobin, Zhu. and Ying, Li designed the research and revise the manuscript. Peng, Shi. and Simin, Xu. wrote the manuscript. Ying, Li. and Liying, Wang. analyzed the data. Yalun, Wu. and Ziling, Yang made the formal analysis. All authors have read the final manuscript and approved it for publication.

## CONFLICT OF INTEREST STATEMENT

The authors declare no conflict of interest.

## ETHICS APPROVAL

No animals or humans were involved in this study.

## Data Availability

The data that support the findings of this study are available on request from the corresponding author. The data are not publicly available due to privacy or ethical restrictions. This manuscript does not generate any new code or data. Supplementary materials (graphical abstract, slides, videos, Chinese translated version and update materials) may be found in the online DOI or iMeta Science http://www.imeta.science/imetaomics/.
